# Effects of Endocrine Disruptor Compounds, Alone or in Combination, on Human Macrophage-Like THP-1 Cell Response

**DOI:** 10.1371/journal.pone.0131428

**Published:** 2015-07-02

**Authors:** N. Couleau, J. Falla, A. Beillerot, E. Battaglia, M. D’Innocenzo, S. Plançon, P. Laval-Gilly, A. Bennasroune

**Affiliations:** 1 Université de Lorraine, CNRS UMR 7360, Laboratoire Interdisciplinaire des Environnements Continentaux (LIEC), IUT Thionville-Yutz, Espace Cormontaigne, Yutz, France; 2 IUT Thionville-Yutz, Impasse Alfred Kastler Espace Cormontaigne, Yutz, France; 3 Université de Lorraine, CNRS UMR 7360, Laboratoire Interdisciplinaire des Environnements Continentaux (LIEC), Campus Bridoux—8, Metz, France; 4 Calcium Signaling and Inflammation Group, Life Sciences Research Unit, University of Luxembourg, Luxembourg, Luxembourg; University of Missouri, UNITED STATES

## Abstract

The aim of the present study was to evaluate the immunological effects on human macrophages of four endocrine disruptor compounds (EDCs) using the differentiated human THP-1 cell line as a model. We studied first the effects of these EDCs, including Bisphenol A (BPA), di-ethylhexyl-phthalate (DEHP), dibutyl phthalate (DBP) and 4-tert-octylphenol (4-OP), either alone or in combination, on cytokine secretion, and phagocytosis. We then determined whether or not these effects were mediated by estrogen receptors via MAPK pathways. It was found that all four EDCs studied reduced strongly the phagocytosis of the differentiated THP-1 cells and that several of these EDCs disturbed also TNF-α, IL-1 β and IL-8 cytokine secretions. Furthermore, relative to control treatment, decreased ERK 1/2 phosphorylation was always associated with EDCs treatments—either alone or in certain combinations (at 0.1 μM for each condition). Lastly, as treatments by an estrogen receptor antagonist suppressed the negative effects on ERK 1/2 phosphorylation observed in cells treated either alone with BPA, DEHP, 4-OP or with the combined treatment of BPA and DEHP, we suggested that estrogen receptor-dependent pathway is involved in mediating the effects of EDCs on human immune system. Altogether, these results advocate that EDCs can disturb human immune response at very low concentrations.

## Introduction

The endocrine system is composed of glands that secrete hormones produced in the body to regulate the activity of cells or organs. Hormones control growth, development, and reproduction as well as the electrolyte composition of body fluids and the metabolism of body [1]. According to European Union, Endocrine Disruptors (ED) are exogenous substances that cause adverse health effects in an intact organism, or its progeny, secondary to changes in endocrine function. Endocrine Disruptor Compounds (EDCs) regroup a large variety of substances such as those used in multiple industrial processes, including solvents/lubricants (polychlorinated biphenyls (PCBs)), plastics (Bisphenol A (BPA) and phthalates), pesticides (DichloroDiphenylTrichloroethane (DDT)) or pharmaceuticals (such as Estradiol (E2)) [2].

EDCs exert their effects differently according to the developmental stages of the affected organisms [2, 3]. Furthermore, a possible latency in the mode of action is another feature of EDCs and exposures during critical developmental period could represent “the basis for adult diseases” [4]. In fact, effects can occur with a lag after exposure [5]. For example, an exposure during early development can induce several effects in adulthood [3]. Another major aspect of exposure to EDCs is related to transgenerational epigenetic effects. For example, Anway et *al*. have observed a persistence of effects along generations after exposure of female rats to several EDCs such as vinclozolin or methoxychlor [6].

Several studies have shown that EDCs directly affect innate immune system. For instance, we observed altered immune response in human macrophages after 4-nonylphenol and diisononylphthalate exposure [7]. Ohnishi *et al*. showed also that some agrochemicals and resin-related chemicals could potentially inhibit macrophage function [8]. In addition, Roy *et al*. found that maternal exposure to BPA modulates innate immunity in adult offspring but not adaptative immune responses to influenza A virus infection in mice [9]. Another work by Watanabe *et al*. showed that BPA enhances neutrophilic maturation of the leukocytes through estrogen receptor-independent pathway, suggesting that BPA affects the innate immunity of mammals [10].

Epidemiological studies have also uncovered the possible link between EDCs and immune disorders; for example, the exposure to phthalates is associated with increased risks to develop allergies and asthma, even though the lack of accurate exposure information limits the interpretation [11].

Since only a rather limited number of works have been devoted to study the effects of EDCs on immune system and the fact that in the human environment EDCs are present as mixtures, we initiated the present work to better understand the direct individual effects of four EDCs on human immune cells, studying these EDCs alone as well as in combination under a wide concentration range.

BPA is an organic compound produced in industrial scale principally for the production of polycarbonate plastics and epoxy resins [12]. These compounds are thus found in a wide range of products such as plastic bottles, papers, food packaging, paints, flaming-retardants compounds [13]. Many previous studies have suggested that BPA has endocrine disruptor effects. For example, in human BPA appears to impair testosterone production in fetal testis and in rodents fetal and perinatal exposures to environmentally relevant doses of BPA can adversely affect the physiological function of endocrine pancreas, mammary gland and reproductive tract [14]. BPA also disturbs the biology of immune cells and human exposure to BPA seems to play a significant role in the initiation or the enhancement of inflammatory response [15].

Phthalate esters constitute another class of EDCs used often to increase the flexibility and workability of polymers [16]. These EDCs can disturb male reproductive system and may be associated to the malformations of the epididymis and vas deferens, and to cryptorchidism) [17, 18]. Two of the five most used phthalates esters are di-(2-ethylhexyl) phthalates (DEHP) and dibutyl phthalate (DBP) [19]. DEHP and DBP are used mainly in PVC (PolyVinyl Chloride)-containing products (such as toys, food packaging, shower curtain, clothes, medical equipments, cars accessories) but are also used in inks, solvents, perfumes or nail polish [19–21].

Octylphenol is an organic compound used mainly in the synthesis of resins and detergents [22]; it is thus used for the fabrication of paper, textiles, pesticides, personal care products etc [23]. This compound is an estrogenic disruptor and is often detectable in environments such as in rivers, wastewater effluents or potable water [2, 22, 23].

Since these chemicals are widely used in industry, human exposure to these EDCs is ubiquitous, organisms are constantly exposed to complex mixtures of these EDCs which simultaneously affect different organ systems (endocrine or other systems), making it thus rather difficult to study the specific endocrine disturbances produced by individual agents [24]. The aim of this study is to determine the effects of these four EDCs, alone and in combinations, on specific immune system related functions, including phagocytosis capacity and cytokine secretion. We investigated possible differences in MAPK (Mitogen-Activated Protein Kinase) activation caused by the EDCs and determined whether such differences were mediated by their effects on estrogen receptor to address the molecular mechanisms of these chemicals on immune targets.

We used as a model for our experimentations the THP-1 monocytic cell line after differentiation with phorbol-12-myristate-13-acetate (PMA). THP-1 cells were isolated from the blood of a one-year human boy with an acute monocytic leukemia. This myeloid monocyte human cell line can be used after differentiation as a macrophage model [7, 25, 26]. THP-1 retains the capacity of IL-1 secretion and phagocytosis [26, 27].

In the present work, we showed that EDCs are able to affect some immune response parameters in macrophage-like human THP-1 cells through the involvement of Estrogen Receptor-dependent ERK1/2 phosphorylation.

## Materials and Methods

### Chemicals

Lipolysaccharides (LPS) from Escherichia coli 0111:B4, phorbol 12-myristate 13-acetate (PMA), fetal bovine serum (FBS), Bisphenol A, di-ethylhexyl-phthalate, dibutylphthalate, 4-tert-octylphenol and thiazolyl blue tetrazolium bromide (MTT) were purchased from Sigma (Saint Quentin en Fallavier, France). RPMI medium, L-glutamine (200 mM) and penicillin-streptomycin solution (10,000 U/ml, 10,000 μg/ml) were purchased from Invitrogen (Cergy Pontoise, France). Structures of EDCs tested in the present work are shown in S1 Fig.

### Cell culture, differentiation and exposure of THP-1 cells to EDCs

THP-1 cell line was obtained from the American Type Culture Collection (Rockville, MD). Cells were cultured in RPMI 1640 medium supplemented with 10% heat-inactivated FBS, 2 mM L-glutamine, 50 U/mL penicillin, and 50 g/mL streptomycin (defined as culture medium afterwards) in a humidified 5% CO_2_ atmosphere. Medium was changed every 3 days to ensure constant cell growth [27].

Cells were differentiated into adherent macrophage-like cells by culturing with 5 ng/mL PMA for 48 h prior to experiments as proposed by Park and colleagues [24].

Differentiated cells were exposed to EDCs alone or in combination (BPA, DBP, DEHP, 4-OP) each at 0.001, 0.1, 1 or 10 μM for 24 hours. Cells were then stimulated by LPS at 10 ng/ml for 24 hours before studying cell viability, phagocytosis capacity, cytokine secretion and MAP kinase activity.

### Cell viability

Cytotoxicity of EDCs on cells were assessed by MTT (3-(4, 5-dimethylthiazol-2-yl)-2, 5-diphenyl tétrazolium bromide) assay. MTT assay is a viability test based on the ability of only viable cells to reduce MTT (yellow in solution) into formazan (dark blue product) [28, 29]. After incubation of differentiated THP-1 cells with EDCs, alone or in combination (0.001, 0.1, 1, 10 μM), or with DMSO vehicle (0.1% final concentration in culture medium) in 96-well plates for 24 h, cells were washed twice with medium and incubated during 4 h at 37°C with 100 μL of 5 mg MTT /mL resuspended in PBS. Resulting crystals were dissolved by adding to the wells an equal volume of SDS/DMF extraction buffer (20% Sodium Dodecyl Sulfate (w/v) and N, N-dimethylformamide; v/v). Finally, absorbance was measured at 570 nm using a microplate spectrophotometer (BioTek PowerWave XS; VWR, Strasbourg, France). The cell viability value was calculated as a percentage of the absorbance obtained from non-exposed (vehicle alone) cells.

### Phagocytosis assay

THP-1 cells were differentiated into macrophages as described above and grown on polylysine-coated glass coverslips (1,000,000 cells per well in twelve-well plates). Cells were then exposed to EDCs alone or in combination (0.001, 0.1, 1, 10 μM) or to DMSO vehicle (0.1% final concentration in culture medium) for 24 h. Cells were washed with medium and incubated with 1 μm-diameter fluorescein isothiocyanate (FITC)-latex beads (L 1030; Sigma-Aldrich) dispersed in culture medium during 6 h (final *ratio* of 1:50 (cell: beads)). Cells were then rinsed with PBS and subsequently fixed with 4% paraformaldehyde (w/v) for 10 min. After extensive washing with PBS, coverslips were mounted in a water-soluble and non-fluorescent mounting medium (Aqua-Poly/Mount, Tebu-bio). Three hundred cells for each exposure condition were observed and phagocytosis index was calculated as follows: (number of cells ingesting at least one bead*100) /number of total cells. All cultures for each set of measurements were done in triplicate.

### Confocal Laser Scanning Microscopy (CLSM)

Phagocytosis of FITC-latex beads by differentiated THP-1 cells were observed with a laser scanning microscope (LSM 510; Carl Zeiss, Thornwood,NY) equipped with a Plan-Apochromat 63 X oil immersion lens (numerical aperture 1.4) or a C-Apochromat 40 X lens (numerical aperture 1.2).

### Western blotting

Before incubation under different experimental conditions, cells were treated or not with ICI-182780 (ICI) (1μM) dissolved in culture medium for 15 minutes. Then, cells were washed with ice-cold phosphate-buffered saline (PBS) and harvested in lysis buffer [30 mM HEPES, pH 7.6, 30 mM NaCl, 1% Nonidet P-40 (vol/vol), 10% glycerol (vol/vol), 50 mM NaF, 10 mM Na pyrophosphate] supplemented with protease inhibitors (Roche Diagnostics, Indianapolis, IN) and 5 mM Na orthovanadate. Cell lysates were cleared by centrifugation at 14,000 g for 5 min at 4°C. Proteins in total lysates were assayed before SDS-PAGE, using the BCA protein assay kit (Pierce Chemical, Rockford, IL) with bovine serum albumin as a standard. Protein aliquots (20 μg) were applied to a 12% SDS-PAGE. After transfer onto nitrocellulose membrane (Whatman, Maidstone, United Kingdom), blots were blocked overnight with 4% Bovine Serum Albumin (BSA) in Tris-buffer saline, 0.1% Tween 20 and then incubated for 2 h with primary antibody: mouse anti-estrogen receptor alpha antibody (1:50 dilution, clone 6F11; Abcam), mouse anti-phosphorylated ERK1/2 antibody (1:2000 dilution, reference 9106; Cell Signaling Technology), rabbit anti-ERK total antibody (1:5000 dilution, reference 9216; Cell Signaling Technology) or mouse anti-beta actin antibodies (1:16000 dilution, reference 3700; Cell Signaling Technology). The membranes were then washed and incubated with secondary antibody for 1h: anti mouse IgG HRP-linked antibody (1:2000 dilution, reference 7076; Cell Signaling Technology) or anti rabbit IgG HRP-linked antibody (1:2000 dilution, reference 7074, Cell Signaling Technology).

In some experiments, membranes were stripped of antibody (Restore Western blot stripping buffer; Pierce Chemical), and reprobed with a different one. The bands were visualized using a substrate kit (Supersignal West Dura; Pierce Chemical), according to the manufacturer’s instructions, and visualized using ChemiDoc (Bio-Rad). Quantitative results were obtained by using Quantity One software (Bio-Rad).

### Quantitative measurements of cytokines

TNF-α, IL-1β and IL-8 levels in the isolated media samples were immediately quantified using ELISA kits (BD Biosciences, Le Pont de Claix, France).

### Statistical methods

Results are expressed as means ± SD. The significance of differences between groups was determined using SIGMA STAT v.3.5 by one-way ANOVA and Tukey’s test. A significant difference between two values was accepted at p < 0.05.

## Results

### Effect of EDCs on THP-1 cell viability

We first checked whether EDCs could affect cell viability within our experimental conditions. No significant alteration in PMA-differentiated THP-1 cell viability was observed after 24 h of exposure to selected EDCs alone or in combination whatever the conditions (BPA, DBP, DEHP, 4-OP, BPA+DEHP, BPA+DBP and 4-OP+DEHP at 0,001–10 μM) in comparison to control (DMSO vehicle, 0.1% final concentration in culture medium). THP-1 cells exposure to these molecules alone or in combination induced a decrease in cell viability always lower than 10% (data not shown).

Furthermore, when LPS solution (10 ng/ml) was provided to differentiated THP-1 cells during a subsequent 24 h-incubation, there was only a slight unsignificant decrease in cell viability (corresponding to less than 10%) whatever the previous endocrine disruptor exposure (data not shown).

### Effect of EDCs on differentiated THP-1 cells phagocytosis capacity

**Fig 1:**
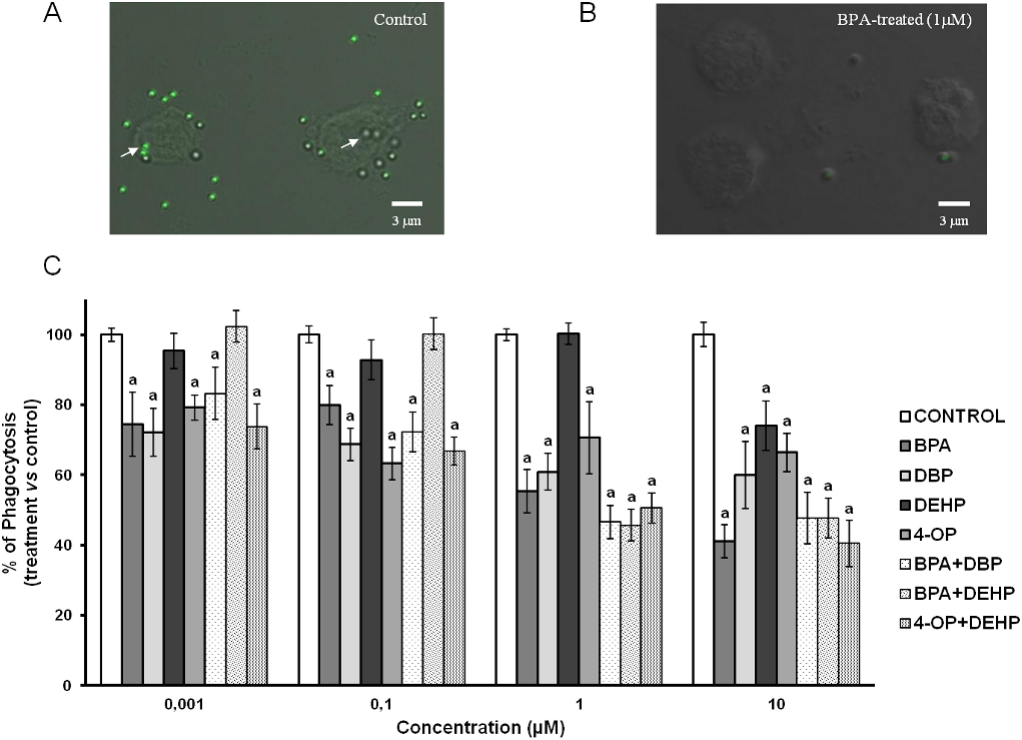
Effect of EDCs alone and in combination on phagocytosis by differentiated THP-1 cells. Differentiated THP-1 cells (PMA 5 ng/ml for 48 hours) were exposed to different concentrations (0.001, 0.1, 1 and 10 μM) of BPA, DEHP, DBP, 4-OP or a combination of both for 24 hours. Culture medium was replaced by fresh medium containing 10 ng/ml of LPS for a subsequent 24 hours of incubation. Panels A and B depict confocal laser microscopy analysis of phagocytosis of FITC-latex beads by untreated differentiated THP-1 cells (A) or by differentiated THP-1 cells exposed to BPA (B). Arrows show examples of phagocytized FITC-beads. Panel C represents the effects of BPA, DEHP, DBP, 4-OP and a combination of both (BPA + DEHP, BPA + DBP, 4-OP + DEHP) on phagocytosis of latex beads by differentiated THP-1 cells. Values are means ± SD (N = 3). Each experiment has been performed three times and in triplicate. a: *vs* control (One-way ANOVA, Tukey post test, p<0.05).

The ability of differentiated THP-1 cells to phagocytize FITC-latex beads was evaluated by analyzing by CLSM THP-1 cells exposed or not to EDCs tested alone or in combination (respectively Fig 1A and 1B). Non-exposed cells showed a phagocytosis capacity of 82% (data not shown).

A concentration-dependent BPA-mediated inhibition of the phagocytosis capacity was observed, with 26.6, 20.1, 44.7 and 59% reduction in comparison to control (DMSO vehicle, 0.1% final concentration in culture medium) after exposures to this compound at respectively 0.001, 0.1, 1 and 10 μM (Fig 1C). These inhibitory effects were found to be significant from 0.001μM of BPA.

DEHP had no effect on phagocytosis capacity of differentiated THP-1 cells except after an exposure at 10 μM which induced a decrease of 24.9% in comparison to control.

A combination of BPA and DEHP induced significant decreases of 54.4 and 52.2% respectively at 1 and 10 μM.

THP-1 cells exposure to DBP reduced their phagocytosis capacity. These significant reductions were respectively 27.8, 33.3, 39.1 and 40.4% in comparison to control for 0.001, 0.1, 1 and 10 μM DBP (Fig 1C). The combination of BPA and DBP (0.001, 0.1, 1 and 10 μM) induced significant decreases in phagocytosis capacity of respectively 16.8, 27.7, 53.4, 52.2% in comparison to control.

4-OP (0.001, 0.1, 1 and 10 μM) led to significant decreases in the phagocytosis capacity of respectively 20.8, 36.4, 29.4 and 33.6%.

A treatment with a combination of 4-OP and DEHP (0.001, 0.1, 1 and 10 μM) induced significant inhibitions of this immune response parameter in a concentration-dependent manner, with a phagocytosis index of respectively 26.2, 33.2, 49.4 and 59.5% in comparison to control.

### Effects of EDCs on TNF-α secretion by differentiated THP-1 cells

**Fig 2:**
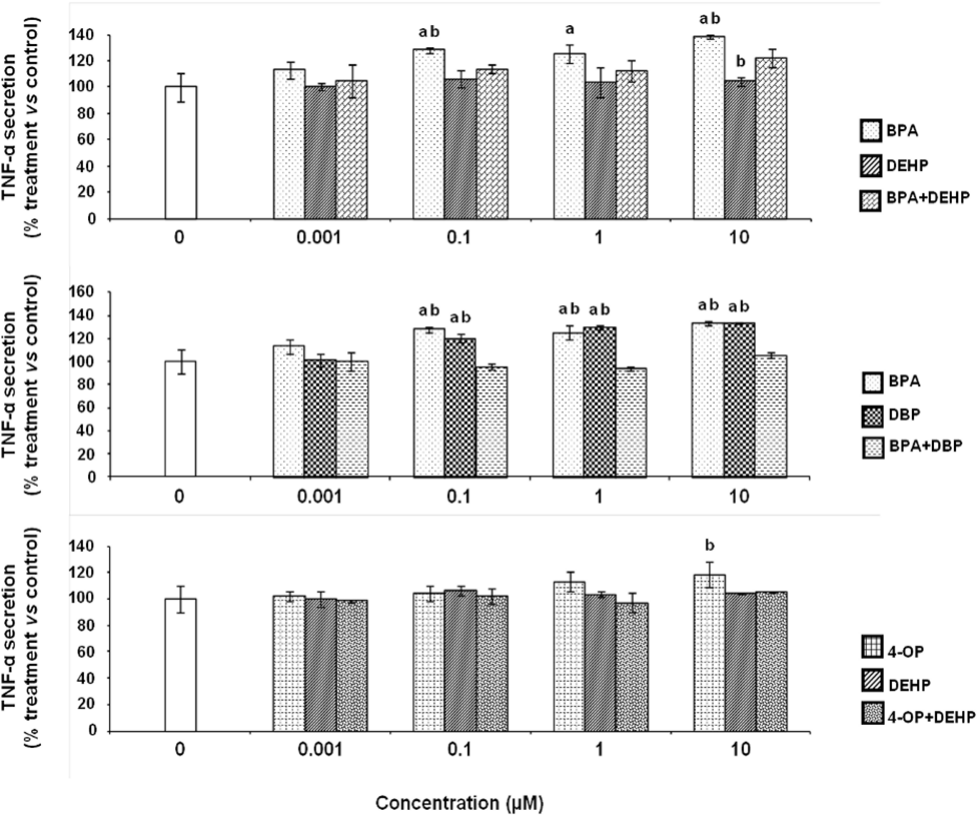
Effect of EDCs alone and in combination on the secretion of TNF-α by differentiated THP-1 cells. Differentiated THP-1 cells were exposed to BPA, DEHP, DBP 4-OP or a combination of both for 24 hours. Culture medium was replaced by fresh medium containing 10 ng/ml of LPS for a subsequent 24 hours of incubation. TNF-α concentrations were determined using a sandwich ELISA test. Values are means ± SD (N = 3). Each experiment has been performed three times and in triplicate. a: *vs* control (Tukey’s test, p<0.05); b: *vs* combination (One-way ANOVA, Tukey post test, p<0.05).

We next evaluated the effects of EDCs on cytokine secretion in PMA-differentiated THP-1 cells. The basal level of TNF-α secretion is 1952 ± 127 pg/mL which is higher than the detection limit (2 pg/mL). Exposure of differentiated THP-1 cells to BPA (0.1, 1 and 10 μM) significantly induced a level of TNF-α secretion which was respectively 28.1, 25.4 and 38.6% higher in comparison to control (cells in culture medium treated with LPS and DMSO vehicle, 0.1% final concentration) (Fig 2). DBP (0.1, 1 and 10 μM) also induced a significant increase in TNF-α secretion respectively 20.2, 29.9 and 33.7% higher than control. The other xenobiotics, alone or in combination (DEHP, 4-OP, BPA+DBP, BPA+DEHP and 4-OP+DEHP), did not significantly modify TNF-α secretion level whatever the concentrations in comparison to control level (Fig 2).

### Effects of EDCs on IL-1 β secretion by differentiated THP-1 cells

**Fig 3:**
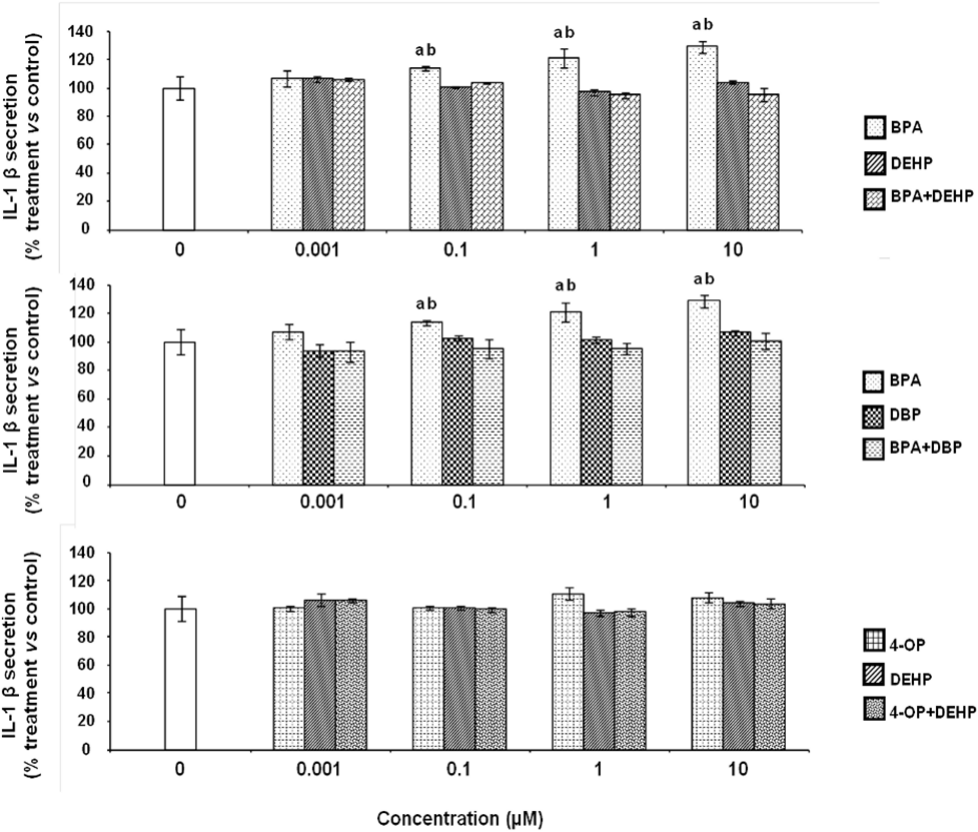
Effect of EDCs alone and in combination on the secretion of IL-1β by differentiated THP-1 cells. Differentiated THP-1 cells were exposed to different concentrations of BPA or DEHP or DBP or 4-OP or a combination of both for 24 hours. Culture medium was replaced by fresh medium containing 10 ng/mL of LPS for a subsequent 24 hours of incubation. IL-1β concentrations were determined using a sandwich ELISA test. Values are means ± SD (N = 3). Each experiment has been performed three times and in triplicate. a: *vs* control (Tukey’s test, p<0.05); b: *vs* combination (One-way ANOVA, Tukey post test, p<0.05).

The basal level of IL-1β secretion is 191 ± 14 pg/mL which is higher than the detection limit (0.8 pg/mL). BPA (0.1, 1 and 10 μM) induced IL-1 βlevel in the medium which was respectively 17.6, 21.1 and 28.7% higher than the level obtained for control cells (cells in culture medium treated with LPS and DMSO vehicle, 0.1% final concentration). No significant modification of IL-1 β secretion was observed with the other EDCs, whatever the concentrations and exposure conditions (EDCs alone or in combination) (Fig 3).

### Effects of EDCs on IL-8 secretion by differentiated THP-1 cells

**Fig 4:**
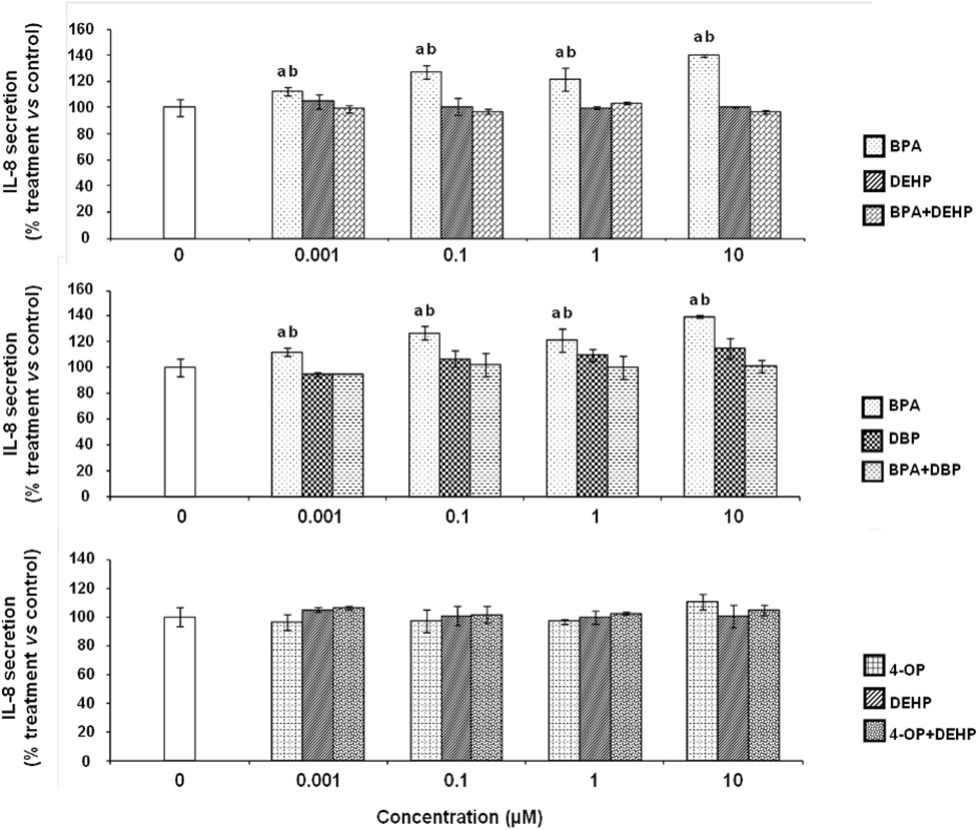
Effect of EDCs alone and in combination on the secretion of IL-8 by differentiated THP-1 cells. Differentiated THP-1 cells were exposed to different concentrations of BPA, DEHP, DBP, 4-OP or a combination of both for 24 hours. Culture medium was replaced by fresh medium containing 10 ng/mL of LPS for a subsequent 24 hours of incubation. IL-8 concentrations were determined using a sandwich ELISA test. Values are means ± SD (N = 3). Each experiment has been performed three times and in triplicate. a: *vs* control (Tukey’s test, p<0.05); b: *vs* combination (One-way ANOVA, Tukey post test, p<0.05).

The basal level of IL-8 secretion is 393 ± 25 pg/mL which is higher than the detection limit (0.8 pg/mL). BPA (0.001, 0.1, 1 and 10 μM) induced IL-8 secretion in the medium to a level which was respectively 12.2, 27.1, 21.4 and 39.8% higher than the level obtained for control cells (cells in culture medium treated with LPS and DMSO vehicle, 0.1% final concentration). No significant modification in the secretion of IL-8 was observed with the other tested EDCs whatever the concentrations and exposure conditions (alone or in combination) (Fig 4).

### Effects of EDCs on estrogen receptor expression and signaling

**Fig 5:**
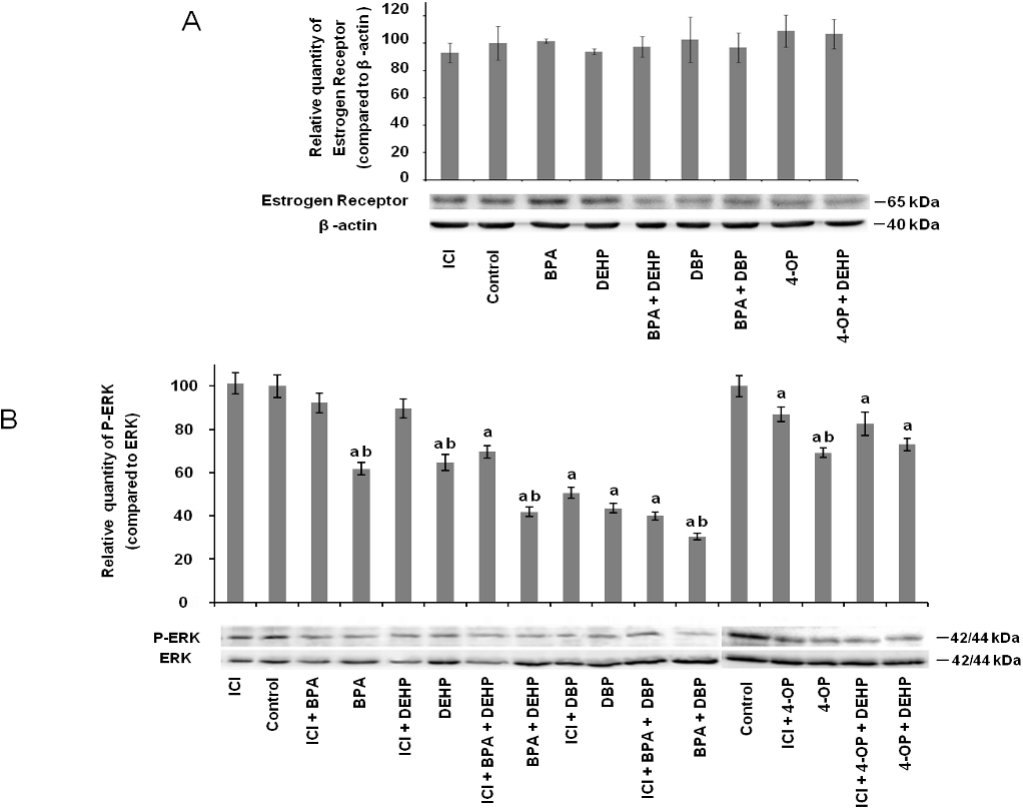
Effect of EDCs alone and in combination on estrogen receptor protein expression and signaling. (A) Differentiated THP-1 cells (PMA 5 ng/ml for 48 hours) were exposed to ICI-182780 (1 μM), BPA, DEHP, BPA + DEHP, DBP, BPA + DBP, 4-OP or 4-OP + DEHP (each EDC at 0.1 μM) for 24 hours. Culture medium was replaced by fresh medium containing 10 ng/mL of LPS for a subsequent 24 hours of incubation. Whole cell lysates (20 μg) were separated by SDS-PAGE (10%) and immunoblotted with the anti-estrogen receptor antibody as described in Materials and Methods. The same blot was stripped and immunoblotted with control antibody (anti-β-actin). Histograms represent the relative quantifications of ERα which were performed in comparison with β-actin. Values in non-treated cells are taken as 100%. Each experiment has been performed four times. Values are means ±SD (N = 4). a: *vs* control (One-way ANOVA, Tukey post test, p<0.05). (B) Differentiated THP-1 cells (PMA 5 ng/ml for 48 hours) were exposed to ICI-182780 alone and in combination with BPA, DEHP, BPA + DEHP, DBP, BPA + DBP, 4-OP, 4-OP + DEHP for 24 hours. Culture medium was replaced by fresh medium containing 10 ng/mL of LPS for a subsequent 24 hours of incubation. Each EDC was also tested without ICI. EDCs and ICI were used at 0.1 μM and 1 μM respectively throughout this experiment. Whole cell lysates (20 μg) were separated by SDS-PAGE (10%) and immunoblotted with the anti-phospho-ERK antibody as described in Materials and Methods. The same blot was stripped and immunoblotted with anti-ERK antibody. Histograms represent the relative quantifications of phospho-ERK which were performed in comparison with ERK. Values in non-treated cells are taken as 100%. Each experiment has been performed three times. Values are means ±SD (N = 3). a: *vs* control (One-way ANOVA, Tukey post test, p<0.05); b: *vs* exposure to corresponding EDCs after treatment by ICI (One-way ANOVA, Tukey post test, p<0.05).

In order to determine if the effects of EDCs are mediated through estrogen receptor signaling, we investigated some functional aspects of these proteins expressed in differentiated THP-1 cells. For this purpose, we evaluated the effects of cell exposures to tested pollutants (BPA, DEHP, BPA+DEHP, DBP, BPA+DBP 4-OP and 4-OP+DEHP) on estrogen receptor alpha (ERα) expression and on activation level of one of the most important kinase in its signaling pathway i.e. ERK 1/2 MAPK. Protein expression level was studied by Western blot analysis of cells extracts from untreated and 0.1 μM EDC-treated THP-1 cells using antibodies directed against ERα, as well as specific antibodies directed towards the phosphorylated form of ERK 1/2 (Fig 5).

All treatments with selected EDCs (alone or in combination) but also with the ER antagonist ICI-182780 (ICI) did not affect ERα expression level in differentiated THP-1 cells in comparison to untreated cells (Fig 5A). EDCs alone or in combination induced a significant decrease in ERK 1/2 phosphorylation in comparison to control cells (DMSO vehicle, 0.1% final concentration in culture medium) (Fig 5B). These ERK 1/2 phosphorylation levels were about 38.2, 35.3, 58.1, 56.5, 69.6, 30.8 and 27%, lower than in control cells after treatments with respectively BPA, DEHP, BPA+DEHP, DBP, BPA+DBP, 4-OP and 4-OP+DEHP. No significant modification of the P-ERK/ERK ratio was observed in cells treated with the receptor antagonist ICI. Finally, ICI treatment with BPA, DEHP, (BPA+DEHP), (BPA+DBP) and 4-OP induced an increase in relative ERK 1/2 phosphorylation level in comparison with cells exposed to corresponding EDCs (Fig 5B). This last result suggests that this estrogen receptor antagonist is able to reduce the effects of BPA, DEHP and 4-OP on ERK 1/2 phosphorylation.

## Discussion

The endocrine disruptors selected in the present study are xenoestrogens which can be found in the environment and can induce adverse effects on endocrine system of several organisms including human. Nevertheless, EDCs are also able to disturb other systems and induce various effects such as obesity, behavior change and cancer [30–32]. Since EDCs have the ability to affect systems other than the endocrine system, we assess in the present work direct effects of bisphenol A, dibutyl phthalate, di-etylhexyl-phthalate and 4-tert-octylphenol-ubiquitous chemical species present in many consumers products- on immune human system, using THP-1 cells. To this end, we exposed differentiated THP-1 cells to these EDCs in order to gain further insights into the mechanism of toxicity of these chemicals on macrophages and to better define the potential role of macrophages in the secretion of pro-inflammatory cytokines in response to EDCs. THP-1 cell line, used in the present study, is considered as a macrophage-like model [25, 33–35].

After exposure of differentiated THP-1 cells to selected EDCs alone or in combination (0, 0.001, 0.1, 1 and 10 μM) during 24 h, no significant effect on cell viability was observed whatever the conditions (BPA, DBP, DEHP, 4-OP, BPA+DEHP, BPA+DBP and 4-OP+DEHP). These results are in accordance with those described in previous studies which showed in several cell models that BPA, DBP, DEHP and 4-OP do not alter cell viability at equivalent or higher concentrations than those tested in the present work [36–38]. We thus were able to evaluate the effects of these compounds on human immune response in these experimental conditions. All four EDCs were tested in a range of concentrations between 0.001 and 10 μM, corresponding to 0.228 μg/L—2.28 mg/L for BPA, 0.39 μg/L—3.9 mg/L for DEHP, 0.278 μg/L—2.78 mg/L for DBP, and 0.206 μg/L—2.06 mg/L for 4-OP. Average plasmatic concentrations reported in the literature for these EDCs are all included within these ranges of concentrations. These values are 0.460 and 0.548 μg/L for BPA [39,40], 218 μg/L for DEHP [41], 35.1 and 135.4 μg/L for DBP [41, 42] and 13.8 to 221 μg/L for 4-OP [43]. Some tested concentrations were also higher than the average plasmatic concentrations but remained lower than those tested in previous studies for BPA [44, 45], DEHP [46, 47], DBP [48] and 4-OP [49].

Phagocytosis capacity of THP-1 cells was investigated by evaluating the ability of differentiated THP-1 cells to phagocytize fluorescent latex beads after exposure to selected EDCs (0, 0.001, 0.1, 1 and 10 μM) using CLSM.

Exposure to BPA or DBP alone induced a reduction of phagocytosis, and in a concentration-dependent manner for BPA. The combination of BPA and DBP provoked also a concentration-dependent inhibition of this parameter without any combined effects (in comparison with BPA or DBP alone). Unlike exposures to DEHP or 4-OP alone and to a combination of BPA+DEHP, which altered phagocytosis capacity but not in concentration-dependent manner, the simultaneous exposure to DEHP and 4-OP induced a concentration-dependent reduction of phagocytosis capacity with additive effect of this mixture for the highest concentration. To the best of our knowledge, only few studies have already shown the EDCs capacity to reduce phagocytosis but never with such low exposure concentrations (notably 1 nM and 100 nM in the present work). For example, in a previous work, Bennasroune et *al*. demonstrated an alteration of differentiated THP-1 phagocytosis after exposure to Diisononylphthalate and 4-Nonylphenol, but with higher exposure concentrations (0.2–10 μM) and without any combined effects [7]. The results of the presents work confirm those of this previous study which suggested that mixtures of EDCs did not provoke any synergistic effect on this immune system parameter under these experimental conditions. Two other studies using aquatic organism models leaded by Canesi et *al*. and Cabas et *al*. showed that estrogenic chemicals are able to alter the phagocytic activity of immunocytes (hemocytes) [50, 51]. Even if the models were different, their studies are in accordance with the present work indicating that EDCs can modify the immune response. Thus, it is important to note that relatively few studies have shown that EDCs induced a diminution of phagocytosis capacity by cells from the human immune system at biological relevant concentrations. The recognition, internalization, and degradation of a pathogen are necessary for normal clearance of an infection. Dysregulation in these processes caused by these compounds can be detrimental to the host defense and human health.

Cytokines are involved in several physiological processes and are especially important for regulating inflammatory and immune (innate and adaptive) responses [52]. As cytokines have an important role in initiating responses once a pathogen penetrates the host [53–55], a few research teams have considered that it is necessary to better understand the effects of EDCs or hormones and their mixtures on the inflammatory response of several organisms. For instance, Jin et *al*. have shown that the mRNA levels of different cytokines such as TNF-α, IFN, or IL-1β were affected in zebrafish when exposed to several EDCs such as nonylphenol or to hormone such as 17β-estradiol. Other previous reports showed that EDCs are able to alter the inflammatory response and host defense system against pathogen in models such as mouse macrophage cell line [56]. Therefore, we investigated potential direct effects of several EDCs alone or in combination (BPA, DBP, DEHP, 4-OP, BPA+DEHP, BPA+DBP and 4-OP+DEHP) on LPS-induced cytokine secretion by differentiated THP-1 cells in order to determine the pro-inflammatory effects of these xenobiotics.

We determined LPS-induced IL-1 β, IL-8 and TNF-α cytokine secretion after cell exposures to EDCs alone or in combination. IL-1 β is described as a pro-inflammatory and "alarm" cytokine which is rapidly produced by macrophages in response to inflammatory stimuli and is able to induce the expression of several genes and the synthesis of proteins that in turn, induce inflammation [57]. Il-8, also called CXL8, is a key mediator in neutrophil-mediated acute inflammation due to its chemotactic activity. Several types of cells can produce a large amount of IL-8/CXL8 in response to a wide variety of stimuli, including proinflammatory cytokines, microbes and environmental changes [58]. Regarding the effects of these xenobiotics on IL-1 β secretion, exposures to BPA from 0.1 to 10 μM increased the LPS-induced stimulation of this cytokine. Moreover, BPA increased LPS-induced IL-8 secretion in a concentration-dependent manner but none of the other tested EDCs induced a modification in the secretion of this chemokine. The present study suggests that EDCs such as BPA can affect pro-inflammatory cytokine secretion by differentiated THP-1 macrophage cells. Indeed, to the best of our knowledge, no direct effect of BPA has been previously reported on IL-8 secretion by human macrophage cells. This finding should be examined more closely using biomarkers of cellular damages and complementary assay techniques in order to establish a causal link between BPA exposure and modification of IL-8 and IL-1 β secretions to better understand the mechanisms involved in the direct inflammatory effects of this compound. TNF-α, a pro-inflammatory cytokine, is a key mediator of inflammation [59]. Concerning the effects of tested EDCs on TNF-α secretion, we observed that only exposures to 0.1 to 10 μM of BPA and DBP alone induced increases in the secretion of this cytokine. Our results are in accordance with previous studies which described that cytokine secretion can be modified after exposure to EDCs. Thus, Kuan et *al*. showed an increase of TNF-α secretion of RAW264.7 mouse macrophage cell line after exposure to bisphenol A-glycidyl-methacrylate [60]. Dysregulation of TNF-α secretion by macrophages at local disease sites is associated with development of inflammatory diseases [61]. To our knowledge, our last result suggests for the first time that BPA or DBP may affect microbes-induced inflammation by the alteration of this response, after exposures to very low and biologically relevant concentrations. Surprisingly, no significant modification of TNF-α secretion level was observed after exposures to a combination of BPA and DBP. These results suggested that BPA and DBP effects may be antagonistic in our experimental conditions. To our knowledge, no similar effects of EDCs has been shown on TNF-α secretion. However, in other cell models, it has been described that a mixture of estrogenic chemicals such as 17β-estradiol, genistein and o,p'-DDT exerted an antagonistic activity in an ERα gene reporter system in human breast cancer MCF-7 cells [62]. Futhermore, in another study, the effects of 17β-estradiol, 17α-ethinylestradiol, genistein, bisphenol A, 4-nonylphenol and 4-tert-octylphenol on the proliferation of estrogen-dependent MCF-7 cells were measured by Rajapakse et al. who showed that 4-nonylphenol and 4-tert-octylphenol appeared to be associated with an antagonistic activity which could be explained by differential activation of drug-metabolizing enzymes (e.g., cytochrome P450) or efflux pumps [63].

In order to evaluate if the effects induced by selected EDCs are mediated through estrogen receptor alpha signaling pathway, we investigated the contribution of these xenobiotics on ERα protein expression and on the phosphorylation level of one of the major kinase of this pathway i.e. ERK 1/2 MAPK. We observed that ERα protein expression was not altered at the tested EDC concentrations, while a decrease in ERK 1/2 phosphorylation level was observed. These effects on ERK 1/2 phosphorylation level were reduced once cells were treated with the ERα antagonist ICI-182780 before exposures to BPA, DEHP, 4-OP or to combinations of BPA and DEHP or BPA and DBP, suggesting that these EDCs mediate their effects at least in part through ERα. Our results are in accordance with previous studies suggesting the involvement of EDCs in estrogen receptor signaling pathway. For example, Yoshitake et *al*. showed that some EDCs (such as BPA and several alkylphenols) suppressed LPS-induced nitric oxide production in mouse macrophage cell through an ERα dependent pathway [56]. Another study indicated that exposure of LPS-stimulated THP-1 derived macrophage to 4-nonylphenol and diisonylphthalatate (at 2 μM i.e. higher concentrations than those tested in the present work) altered phosphorylation level of ERK 1/2 through an ERα dependent pathway, without modification of ERα protein expression [7].

Previous studies using several cell or animal models support the involvement of EDCs in the dysregulation of immune homeostasis, but the mechanisms of action remain unclear. The novel effects observed in the present work in our experimental conditions (including notably biological relevant concentrations) could be explained by different processes such as epigenetics effects. For example, the *in utero* and neonatal exposure to BPA and/or phthalates (such as DBP and DEHP) can be associated with DNA hypermethylation/hypomethylation, histone modifications and expression of non-coding RNAs. These epigenetic markers can alter gene expression that may persist throughout a lifetime and these changes will result in adverse health effects such as immune disorders [64]. Another study by Hung et al. suggests that Nonylphenol and 4‑OP may have functional effects on the response of circulating myeloid dendritic cells via, in part, the ER, MKK3/6-p38 MAPK signaling pathway, and histone modifications, with subsequent influence on the T-cell cytokine responses such as TNF-α secretion [65]. Thus, epigenetic regulation could partially explain the several modulations of immune response parameters observed in our study after exposures of differentiated THP-1 cells to EDCs.

In conclusion, the present study showed that the *in vitro* exposure of differentiated THP-1 derived macrophages to selected EDCs disturbs some immune response parameters such as phagocytosis capacity and pro-inflammatory cytokine secretion, by directly affecting the immune cell response. Moreover, these EDCs have the capacity to modulate ERK 1/2 phosphorylation level, which is mediated through estrogen receptor alpha for 4-OP, BPA, DEHP and the mixture of these two last xenobiotics. It would be interesting to confirm these results using rats as an *in vivo* model. It would be also of interest to identify the metabolites of the tested compounds in the present work in order to evaluate the contribution of these metabolites on cell immune response, and to investigate if the effects observed are due to epigenetics regulation.

## Supporting Information

S1 Fig Chemical structures of the EDCs studied. (TIF)
