# Supplementary figures and images for: Effects of Endocrine Disruptor Compounds, Alone or in Combination, on Human Macrophage-Like THP-1 Cell Response
Source: PLoS One. 2015 Jul 2;10(7):e0131428. doi: 10.1371/journal.pone.0131428 (PMC4489735; doi:10.1371/journal.pone.0131428)

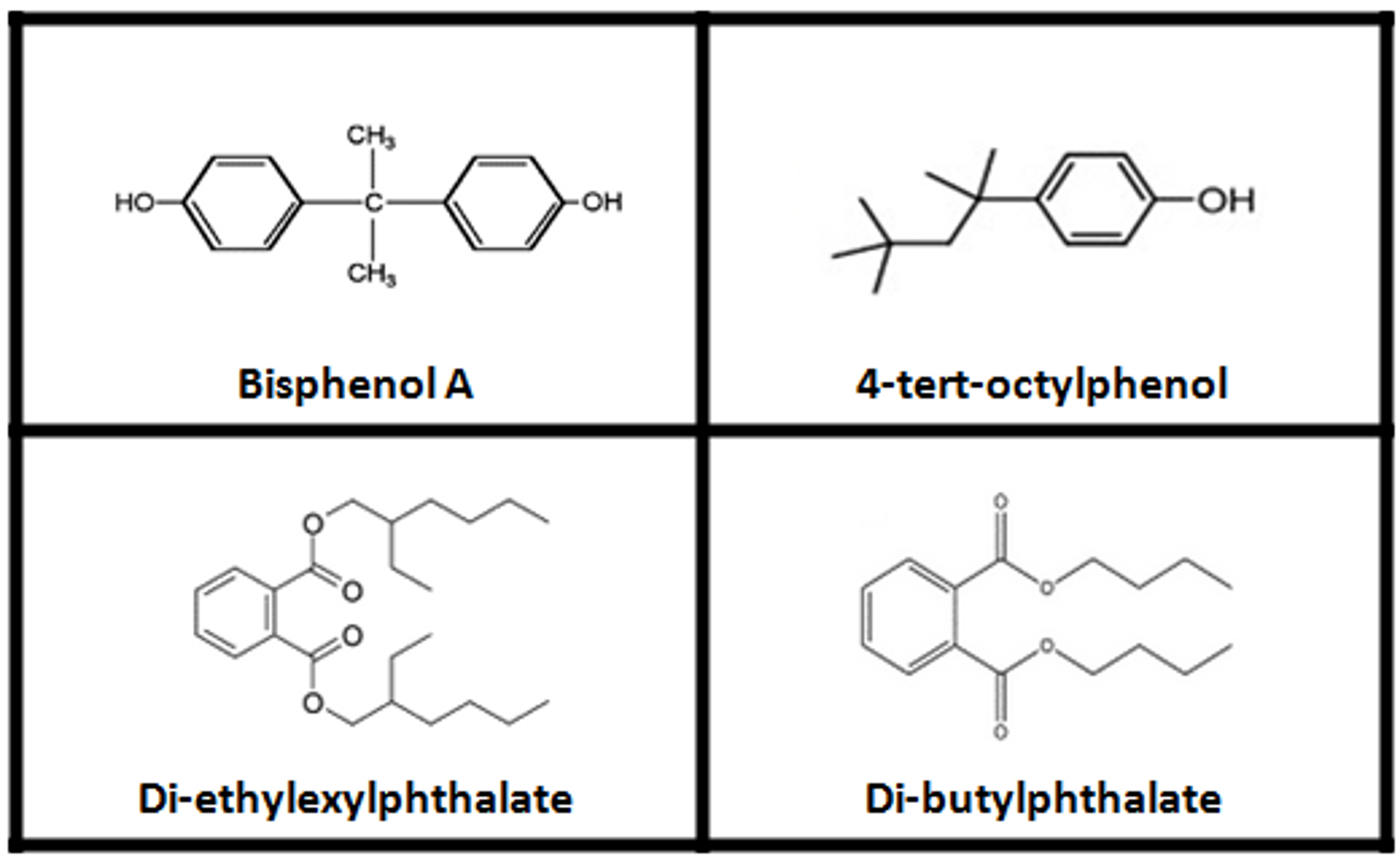

Supplement: S1 Fig — (TIF) [file pone.0131428.s001.TIF]
